# Chitosan Scaffolds as Microcarriers for Dynamic Culture of Human Neural Stem Cells

**DOI:** 10.3390/pharmaceutics15071957

**Published:** 2023-07-15

**Authors:** Yoshiki Ando, Fei-Chien Chang, Matthew James, Yang Zhou, Miqin Zhang

**Affiliations:** 1 Department of Materials Science and Engineering, University of Washington, Seattle, WA 98195, USA; 2 Materials Department, Medical R&D Center, Corporate R&D Group, KYOCERA Corporation, Yasu 520-2362, Shiga, Japan

**Keywords:** human neural stem cells, bioreactor, dynamic culture, scaffolds, chitosan

## Abstract

Human neural stem cells (hNSCs) possess remarkable potential for regenerative medicine in the treatment of presently incurable diseases. However, a key challenge lies in producing sufficient quantities of hNSCs, which is necessary for effective treatment. Dynamic culture systems are recognized as a powerful approach to producing large quantities of hNSCs required, where microcarriers play a critical role in supporting cell expansion. Nevertheless, the currently available microcarriers have limitations, including a lack of appropriate surface chemistry to promote cell adhesion, inadequate mechanical properties to protect cells from dynamic forces, and poor suitability for mass production. Here, we present the development of three-dimensional (3D) chitosan scaffolds as microcarriers for hNSC expansion under defined conditions in bioreactors. We demonstrate that chitosan scaffolds with a concentration of 4 wt% (4CS scaffolds) exhibit desirable microstructural characteristics and mechanical properties suited for hNSC expansion. Furthermore, they could also withstand degradation in dynamic conditions. The 4CS scaffold condition yields optimal metabolic activity, cell adhesion, and protein expression, enabling sustained hNSC expansion for up to three weeks in a dynamic culture. Our study introduces an effective microcarrier approach for prolonged expansion of hNSCs, which has the potential for mass production in a three-dimensional setting.

## 1. Introduction

The demand for human neural stem cells (hNSCs) is substantial in the fields of regenerative medicine and pharmacological development. These versatile cells play a crucial role in medical research and the treatment of challenging diseases such as Alzheimer’s disease, Parkinson’s disease, amyotrophic lateral sclerosis, Huntington’s disease, and spinal cord injury [1,2]. These stem-cell based applications require the administration of tens of millions of purified cells for each patient. hNSCs are commonly grown on 2D plastic cultureware [3,4]. However, conventional 2D culture systems, commonly employed in hNSC growth, have limitations. These include restricted cell productivity due to limited growth area, the absence of microenvironmental cues resembling native tissues, and non-compliance with current good manufacturing practices (cGMP) guidelines, making mass production challenging and costly. As such, researchers have explored the utilization of three-dimensional (3D) culture systems for production of stem cells such as induced pluripotent stem cells (iPSCs), embryonic stem cells (ESCs), and mesenchymal stem cells to address these limitations and better mimic in vivo conditions [5,6].

Despite extensive investigations into 3D culture systems, [7,8], customized technologies specifically targeting the long-term, large-scale production of hNSCs in these settings are yet to be explored. One of the primary obstacles encountered in the 3D production of stem cells is necrosis, primarily caused by the formation of stagnant zones in the system and the presence of large cell clusters. To address these challenges, dynamic culture conditions, those commonly found in bioreactors, have been identified as an effective means of eliminating stagnant zones in 3D cultures. The utilization of dynamic culture conditions may enable the production of a remarkable scale of 10^9^ to 10^12^ high-quality cells per patient [9,10,11,12,13,14].

Although bioreactors are widely utilized for large-scale, 3D production of microorganisms, such as bacteria or cells, in dynamic environments, their implementation for hNSCs presents significant challenges. Ideally, bioreactors should facilitate efficient nutrient and oxygen exchange essential for cell proliferation, metabolite removal, and abundant cell yield. However, there is currently a lack of scalable, dynamic cell culture methods, as well as bioreactor designs specifically tailored for hNSC production. The dynamic environment in bioreactors generates shear stress, which poses threat to cell viability and can induce unintended cell differentiation [15], and yet the precise impact of shear stress on cell fate remains poorly understood [16,17,18]. Moreover, limited knowledge is available about the ability of bioreactors to maintain undifferentiated hNSC populations over extended periods. Most existing protocols focus on the production of ESCs or iPSCs immediately prior to differentiation [19]. These protocols typically involve initiating the culture with iPSC aggregates in suspension for neural differentiation to generate NSCs and neurons. However, these methods are not suitable for most purified hNSCs derived from patients, as the microenvironment of cell aggregates is not established by hNSCs themselves but rather by a mixed population of differentiating cells that aid in cell development and survival. As a result, patient-derived hNSC clusters exhibit low survival and proliferation rates in suspension cultures [20,21,22]. Although a few studies have explored bioreactor systems for inducing human neural precursor cells in a matrix-free suspension, such approaches have not gained widespread popularity [23,24,25,26]. Matrix-free suspension cell cultures commonly suffer from issues such as spontaneous unwanted differentiation, particularly towards glial cells, as well as necrosis resulting from inadequate nutrition and oxygen supply in the central region of cell aggregates [20]. To address these challenges, matrix-dependent dynamic systems utilizing microcarriers have been proposed as more reliable and applicable to a broader range of cell types [27,28,29].

Microcarriers offer a 3D supportive matrix that enables cell adhesion and proliferation. However, in most hNSC cultures, including microcarriers and dish surfaces, the use of biologically relevant molecules such as Matrigel or laminin is necessary to achieve significant cell adhesion. Unfortunately, these molecules are expensive and do not meet cGMP standards. Therefore, there is a need for an appropriate microcarrier design that addresses these limitations and provides several advantages for culture in bioreactors. Firstly, an ideal microcarrier would allow cells to reside inside the microcarrier, protecting them from shear stress-induced apoptosis, detachment, and spontaneous differentiation. This would enable the utilization of various bioreactors and operating parameters. Secondly, the surface chemistry of microcarriers, whether through a cationic or biomimetic interface, could activate crucial cell surface receptors that promote cell adhesion, survival, and proliferation. Thirdly, the microstructure of microcarriers could regulate cell morphology and distribution, preventing necrosis and controlling the size of cell aggregates. Lastly, microcarriers offer the flexibility to tailor the microenvironment to better accommodate different tissue or cell subtypes by modifying the microcarrier design.

Too meet these criteria, researchers have been investigating porous microcarriers as a potential solution [30,31,32]. Porous materials can be made from metals or ceramics [33]. However, their high specific gravity makes them unsuitable for floating and flowing in a dynamic environment. Therefore, biocompatible polymers such as chitosan, dextran, cellulose, collagen, alginate, poly (lactic-*co*-glycolic acid), polylactide, and polystyrene are considered better candidates for porous microcarrier materials [34,35,36,37,38,39,40,41]. However, some of these materials have limitations such as being xenogenic, lacking the necessary surface chemistry to promote cell adhesion, or being expensive to scale up.

Chitosan, a natural polysaccharide derived from fungi or the shells of crustaceans like crabs or shrimp, is the second most abundant polymeric material after cellulose. It finds applications as a raw material for food, agriculture, and medical equipment industries [42]. Unlike collagen, chitosan does not harbor zoonotic pathogens, and the purification process removes allergenic crustacean proteins, minimizing the risk of allergic reactions in humans [43]. The cationic nature of chitosan along with its structural similarity to glycosaminoglycans enables mammalian cells to adhere to it effectively [36]. Additionally, chitosan exhibits a balanced hydrophilic–hydrophobic nature and possesses antimicrobial properties. Compared to collagen, chitosan has a higher elastic modulus, making it more suitable for maintaining structural integrity in dynamic cultures [44]. Importantly, chitosan has been proven to be compatible with neural tissue regeneration [45], and it has been incorporated into numerous medical implants for nerve repair [46,47,48].

This study introduces porous chitosan scaffolds constructed and optimized as microcarriers for the expansion of hNSCs in dynamic cultures. The scaffolds were fabricated through lyophilization using solutions with varying concentrations of chitosan, with collagen serving as a control. Compression tests and atomic force microscopy (AFM) were conducted to assess the elastic modulus and overall mechanical strength of the scaffolds. The chitosan scaffolds demonstrated excellent stability in the culture medium for a duration of three weeks. By tailoring the pore structure of the scaffolds specific for hNSCs, optimal 3D distribution and infiltration of the cells were achieved while providing protection against shear stress inducted by the dynamic environment. Cell adhesion, proliferation, and multipotency were characterized to identify the most suitable type of scaffolds for culturing hNSCs in a dynamic setting. The study presents these chitosan scaffolds as viable microcarriers capable of scaling up hNSC production in dynamic and well-defined cultures.

## 2. Materials and Methods

### 2.1. Microcarrier Fabrication

All chemicals were purchased from MilliporeSigma (St. Louis, MO, USA) unless otherwise specified. To make chitosan porous scaffolds, chitosan (MW, 50–100 kDa; degree of deacetylation, 95–98%; MarkNature, Fullerton, CA, USA) was dissolved homogeneously in 1 N acetic acid at chitosan concentrations of 0.5 wt% (05CS), 2 wt% (2CS), and 4 wt% (4CS). To make collagen porous scaffolds, collagen was dissolved in 0.5 N acetic acid at 0.5 wt% (05COL). The solutions were cast into a 24-well plate and frozen at −20 °C for 8 h. Then, the frozen scaffolds were lyophilized in a VirTis Virtual Lyophilizer (SP Scientific, Gardiner, NY, USA) for 1–3 days. No crosslinking steps were performed on the scaffolds. The samples were sectioned into 15 mm in diameter and 0.8 mm thickness for cell culture or 3.0 mm thickness for mechanical testing and dried in a desiccator for 24 h. The dried samples were neutralized with a basic solution (NH_4_OH 33% 15 mL, deionized water (DI) 35 mL, ethanol 500 mL) for 30 min and rinsed with DI water to remove ammonia hydroxide and ethanol.

### 2.2. FTIR and ^1^H NMR Spectroscopy

FTIR analysis of chitosan and collagen powders was performed using a Nicolet 5DXB spectrometer (Thermo Scientific, Boston, MA, USA). The polymer powders were analyzed by adding the polymer to a KBr powder. The polymer and KBr mixture were then pressed to form a thin film. The films were analyzed by averaging 32 scans at a resolution of 2 cm^−1^ over a range of 500–4000 cm^−1^. ^1^H NMR analysis was performed to verify the degree of deacetylation of the chitosan scaffold. Chitosan was first dissolved in 2.5% DCl D_2_O at 10 mg/mL. A 300 MHz spectrometer (Bruker Avance III 300, Billerica, MA, USA) was used to acquire the spectra in triplicates at 25 °C. The degree of deacetylation was determined by calculating the integrals of the protons on the H-Acetyl group and the protons on the H2/H6 group. The following equation was then used to determine the degree of deacetylation:

(1) $$DD\left(\%\right)=\left[1-2\left(\frac{I_{H-Acetyl}}{I_{H2/H6}}\right)\right]\times 100$$

where DD (%) is the degree of deacetylation in percent, I_H-Acetyl_ is the integral of the protons on the acetylated group, and I_H2/H6_ is the integral of the protons on the monomer hexose rings.

### 2.3. Microstructure Characterization

The microstructure of dry scaffolds was examined using scanning electron microscopy (SEM). To prepare SEM samples, the scaffolds underwent a series of steps. First, they were neutralized with water and ammonia hydroxide. Then, the scaffolds were sterilized with ethanol. Subsequently, the scaffolds were dried under vacuum conditions. The dried scaffolds were mounted on a carbon-conductive adhesive tape (NEM Tape, Nisshin EM. Co., Ltd. Tokyo, Japan). These were imaged at 5 kV using a TM3000 tabletop SEM (Hitachi High-Tech Corporation, Tokyo, Japan). From SEM images, pore diameters were calculated with ImageJ software Version 1.53k (NIH, Bethesda, MD, USA).

The porosity of scaffolds was measured using a modified isopropanol displacement method. The dry scaffold volume (*V*, cm^3^) and weight (*W*_1_, g) were measured as follows. The scaffolds were fully immersed in 5 mL of isopropanol (*ρ_s_*, 0.786 g/cm^3^) under vacuum for 15 min to expel air from the pores. Saturated scaffolds were weighed (*W*_2_, g) immediately upon removal from isopropanol bath. The volume of the scaffolds did not change after isopropanol saturation. Porosity (%) was defined as the ratio of the volume of isopropanol absorbed by the scaffolds to the volume of the scaffold and calculated as follows (*n* = 10):

(2) $$Porosity\left(\%\right)=\frac{W_{2}-W_{1}/\rho_{s}}{V}\times 100$$

where *V* is the volume of the dry scaffold, *W*_2_ is the weight after removal from the isopropanol bath, *W*_1_ is the initial weight of the scaffold, and *ρ_s_* is the density of isopropanol. The specific surface area of the scaffold was measured using the multipoint Brunauer–Emmett–Teller (BET) method in NOVA 4200e (Quantachrome Instruments, Boyton Beach, FL, USA). The scaffold was prepared and freeze dried again after the final wash. The dry scaffold was degassed at 50 °C for 12 h. Then, with nitrogen as the adsorbate, isotherms were acquired at 77 K. A multipoint BET was then performed on the isotherms to determine the specific surface area of each scaffold.

### 2.4. Mechanical Property

Uniaxial compression tests were carried out to understand the macromechanical properties of the scaffolds using an Autograph Table-TOP Precision Universal Tester AGS-X (SHIMADZU CORPORATION, Kyoto, Japan) at room temperature. Dry scaffolds were compressed at 0.4 mm/min until at least 40% strain. The compressive Young’s moduli of the scaffolds were calculated as the slope of the linear regions of the stress–strain curves within the range of 0–15% strain.

AFM was performed with a Nanosurf FlexAFM (Nanosurf AG, Liestal, Switzerland). A contact AFM probe mode with a spring constant of 0.2 N/m and resonance frequency of 13 kHz was used to obtain the elastic moduli of the scaffolds immersed in PBS.

### 2.5. Degradation Test

The stability of the scaffolds was investigated by testing their degradation. Dry scaffolds were incubated in an Orbital Shaker PSU-10i (SIA Biosan, Riga, Latvia) with 37 °C Neurobasal medium at 100 rpm. After 7, 14, and 21 days of incubation, the samples were removed, rinsed with distilled water, and lyophilized. The remaining mass (%) of each scaffold after the incubation relative to the initial weight was calculated to assess the degradation.

### 2.6. Cell Culture

hNSC-H14 (WB0195) cells, derived from NIH-approved hESC-H14 (WA14), were obtained from the WiCell Research Institute (Madison, WI, USA). Following the WiCell hNSC protocol, the cells were cultured as monolayers on Geltrex-coated tissue culture dishes, and fresh media were supplied every other day. Human recombinant basic fibroblast growth factor (bFGF, Gibco^®^, Thermo Fisher Scientific, Waltham, MA, USA) and epidermal growth factor (EGF) were supplied daily at 20 ng/mL. The hNSC culture medium comprised a Neurobasal medium (Gibco^®^) supplemented with B 27, N2, MEM NEAA, heparin, and GlutaMAX. hNSCs were passaged every 3–5 days and dissociated with Accutase after reaching 90% confluency.

ReNcell VM was obtained from MillporeSigma (St. Louis, MO, USA). Following the manufacturer’s protocol, the cells were cultured as monolayers on laminin (CC095-M, MillporeSigma)-coated culture dishes. Fresh medium (SCM005, MilliporeSigma), bFGF, and EGF at 20 ng/mL were supplied every other day. The cells were passaged every 3–5 days and dissociated with Accutase after reaching 80% confluency. All cells were incubated in a humidified incubator containing 5% CO_2_ at 37 °C.

### 2.7. Cell Proliferation in a Rotational Bioreactor as a Dynamic Culture

One day after cell inoculation, scaffolds with hNSCs were transferred to a 15 mL flat-bottomed tube containing 1 mL of medium per two scaffolds. The samples were shaken at 65 rpm on an orbital shaker in the following culture period.

Cell proliferation was assessed by an alamarBlue assay (MilliporeSigma) after 3, 5, 7, 11, and 15 days of culture. The scaffolds were transferred to a 24-well plate and gently rinsed with PBS. The medium was replaced with alamarBlue reagent (11 μg/mL resazurin in culture medium). After incubation at 37 °C for 2 h, all solutions were transferred to 96-well black well plates to measure the fluorescence intensity at 590 nm with excitation at 560 nm using a SpectraMax M2 microplate reader (Molecular Devices LLC, San Jose, CA, USA).

Cell morphology and viability were monitored using a LIVE/DEAD^TM^ Viability/Cytotoxicity Kit (Invitrogen Corporation, Waltham, MA, USA), following the manufacturer’s protocol. Briefly, viable cells were stained with 1 μM calcein-AM, while dead cells were stained with 4 μM ethidium homodiemer-1 in a 1:1 mixture of culture medium and phosphate buffered solution for 15–30 min. The scaffolds were imaged using a Nikon TE300 inverted microscope (NIKON CORPORATION, Tokyo, Japan).

### 2.8. Flow Cytometry

Single cell suspensions were collected from the scaffolds. A fixable yellow dead stain kit (Invitrogen) was used to identify dead cells. The cells were fixed with 4% paraformaldehyde for 10 min on ice, permeabilized in 1% *v*/*v* Triton-X 100, washed with PhosFlow Perm/Wash Buffer I (BD Biosciences, San Jose, CA, USA), and resuspended in a buffer. Antibodies PE anti-nestin (BDB561230), PerCP-Cy5.5 anti-SOX1 (BDB561549), Alexa Fluor 647 anti-SOX2 (BDB560302), and Alexa Flour 488 anti-PAX6 (BD561664) were added, incubated on ice for 1 h, and washed with a buffer. The cells were resuspended in 200 μL of buffer and stained with DAPI. All samples were analyzed on a LSRII flow cytometer (BD Biosciences), and the data were plotted using FlowJo software Version 9.9.4 (Tree Star Inc., Ashland, OR, USA).

### 2.9. Immunocytochemistry for NSC Marker Proteins

Human neural stem cell ICC kits (Cat. A24354, Thermo Fisher Scientific) were used, following the manufacturer’s instructions. Briefly, scaffolds bearing the cells were rinsed with PBS, fixed with cold 4% paraformaldehyde on ice for 10 min, permeabilized for 10 min, blocked for 1 h, and stained with SOX2 and nestin primary antibody overnight at 4 °C. Secondary antibodies, Alexa Fluor 488 and 555, were added to the scaffolds the next day and the scaffolds were incubated for 1–2 h on ice, washed thrice with PBS, stained with DAPI, and mounted in a ProLong Gold anti-fade reagent (Thermo Fisher Scientific). Fluorescence images were acquired using a Nikon TE 300 inverted microscope.

### 2.10. Statistical Analysis

All results are presented as the mean ± standard deviation (SD). Statistical significance was determined using one-way analysis of variance with Tukey’s multiple comparison test. Significant differences were set at *p*-value < 0.05.

## 3. Results and Discussion

### 3.1. Development of Porous Microcarriers for hNSC Expansion in a Defined Environment Condition

**Figure 1:**
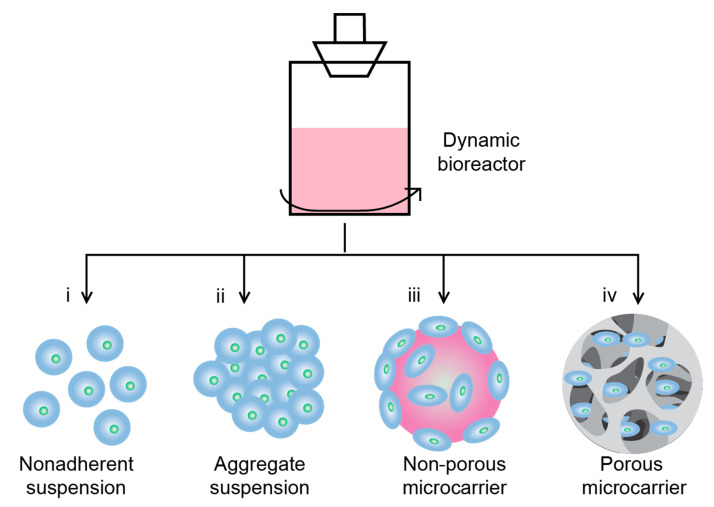
Illustration depicting different culture methods in a dynamic bioreactor, including (i) nonadherent suspension, (ii) aggregate suspension, (iii) nonporous microcarrier, and (iv) porous microcarrier.

Four main culture modes commonly employed in bioreactors are nonadherent (matrix-free) suspension, aggregate suspension, adherent nonporous microcarriers, and adherent porous microcarriers (Figure 1). The utilization of microcarriers in adherent cultures allows for cell adhesion on controlled growth surfaces in dynamic culture conditions. The surface characteristics and microstructure of microcarriers play critical roles in cell quality and yield. In comparison to nonporous microcarriers, the porous design provides the largest growth surface area per volume and desirable interior porous structure to protect cells from shear stress. Therefore, we fabricated porous scaffolds made of chitosan to serve as microcarriers for the expansion of hNSCs in a rotational dynamic bioreactor platform. Chitosan scaffolds with varying weight percentages were utilized to investigate the influence of structural and mechanical properties on cell fate under dynamic conditions. Collagen was used as a control sample due to its well-established role as a scaffold material in tissue engineering [49,50,51]. Although both chitosan and collagen have been applied in various tissue culture substrates, including neural tissues, they possess distinct physical properties and bioactivities on hNSCs [51,52,53,54,55,56].

### 3.2. Scaffold Microstructure Analysis

**Figure 2:**
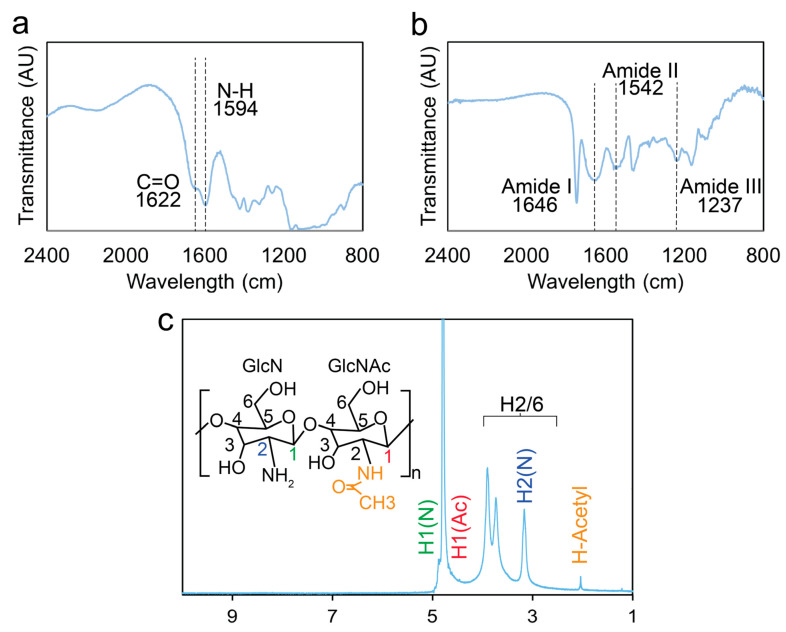
Spectroscopic analysis of the scaffolds. The FTIR spectra acquired (**a**) from chitosan and (**b**) collagen. (**c**) ^1^H NMR spectrum acquired from chitosan.

The microstructure of the scaffolds plays a crucial role in facilitating nutrient cycling and metabolite exchange. To achieve the desired porous structure, the scaffolds were prepared through lyophilization, which involves phase separation during freezing and subsequent sublimation of the solvent. Precise control of nucleation and freezing solvent was employed to establish the desired porosity. Chitosan or collagen was mixed with KBr to form a pellet before FTIR analysis, while chitosan was dissolved in DCl/D_2_O for ^1^H NMR analysis. The FTIR spectra of chitosan and collagen are shown in Figure 2a and Figure 2b, respectively, and exhibited good agreement with those reported previously [57,58]. The ^1^H-NMR spectrum of chitosan, obtained at 300 MHz, 2.5% DCl/D_2_O, 25 °C, displayed the following peaks: 4.80 (s, H-1 of GlcN), 4.50 (s, H-1 of GlcNAc), 3.03 (s, H-2 of GlcN), 2.75–4.15 (m, H-2/6), and 1.95 (s, H-Acetyl of GluNAc), as shown in Figure 2c. The degree of deacetylation was calculated to be 97% using Equation (1), which falls within the range provided by the supplier [59,60].

**Figure 3:**
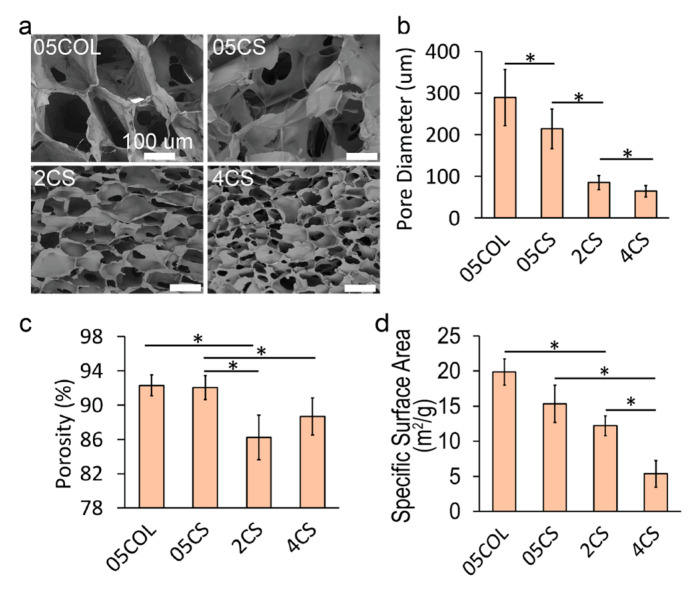
Microstructural analysis of porous scaffolds. (**a**) SEM images of dry porous scaffolds with a scale bar of 100 µm. (**b**) Pore diameters of the scaffolds measured from SEM images (*n* = 65). (**c**) Porosity through the isopropanol displacement method (*n* = 5). (**d**) BET-specific surface area (*n* = 3). All data are presented as the mean ± SD. Statistical significance, indicated by *, was determined using Tukey’s test (*p* < 0.05).

Following lyophilization, the scaffolds were mounted on stubs using carbon adhesive tape for imaging purposes. ImageJ software was utilized for manual measurement of pore diameters. Figure 3a presents SEM images displaying the cross-sections of various scaffolds, including 0.5 wt% collagen (05COL), 0.5 wt% chitosan (05CS), 2 wt% chitosan (2CS), and 4 wt% chitosan (4CS). Lower polymer concentrations resulted in visibly larger interconnected pores. Pore size analysis was performed by measuring the pore diameters from SEM images (Figure 3b). The average pore diameter for 05COL scaffolds was 289 ± 67 μm, similar to commercially available collagen scaffolds [61]. The selected collagen concentration of 0.5 wt% aligns with that in previous studies [62,63,64]. On the other hand, 05CS scaffolds exhibited an average pore diameter of 214 ± 48 μm, significantly different from that of 05COL scaffolds. As chitosan concentration increased, pore diameter decreased, with average values of 85 ± 17 µm and 65 ± 13 µm for 2CS and 4CS scaffolds, respectively. All scaffolds possessed pore diameters suitable for stem cell growth [65], particularly hNSCs [36], providing ample internal surface area for cell proliferation. Moreover, the small pore sizes could limit the growth of hNSC clusters, mechanically preventing the formation of necrotic cores and aiding in the long-term culture and maintenance of hNSCs.

Scaffold porosity was determined using a modified isopropanol displacement method. Briefly, the scaffolds were immersed in isopropanol and subjected to a vacuum to remove air bubbles. Weight measurements were recorded before and after submersion. Porosity (Figure 3c) decreased from 92.0% ± 1.2% to 86.2% ± 2.6% as chitosan concentration increased from 0.5% to 4%. The porosity of 05COL scaffolds was comparable to that of 05CS scaffolds at 92.3% ± 1.4% (*p* > 0.05, not significant). Additionally, specific surface area analysis using BET (Figure 3d) revealed an increase in specific surface area with higher chitosan concentrations: 15.34 ± 2.66 m^2^/g, 12.2 ± 1.41 m^2^/g, and 5.4 ± 1.89 m^2^/g for 05CS, 2CS, and 4CS scaffolds, respectively. Porosity and surface area measurements account for the overall properties of the scaffold, encompassing both the internal intermolecular structures and the visible macropores (50–350 µm in diameter). Collagen and chitosan, being relatively hydrophilic materials, can form nanoscale crystals during freezing and leave voids after sublimation due to solvent (water) bonding in intermolecular spaces. The formation of intermolecular bonds via hydrophobic interactions and hydrogen bonding is more likely in chitosan than in collagen. Chitosan with a high uniform degree of deacetylation is known to exhibit higher crystallinity upon drying [66]. Higher polymer concentrations promote inter- and intramolecular interactions, resulting in reduced nanoscale interaction of polymer chains, which leads to lower material specific area at the nanoscale level and lower porosity in 05CS scaffolds compared to those in 05COL scaffolds.

The microstructure analyses confirmed that the scaffolds met the structural requirements of microcarriers, with highly interconnected pores possessing appropriate diameters to retain media, facilitate nutrient exchange, and provide a larger growth area compared to those of 2D flat surfaces. These characteristics make the scaffolds well-suited for their intended purpose of supporting hNSC expansion in a dynamic culture environment.

### 3.3. Mechanical Properties of the Scaffolds

**Figure 4:**
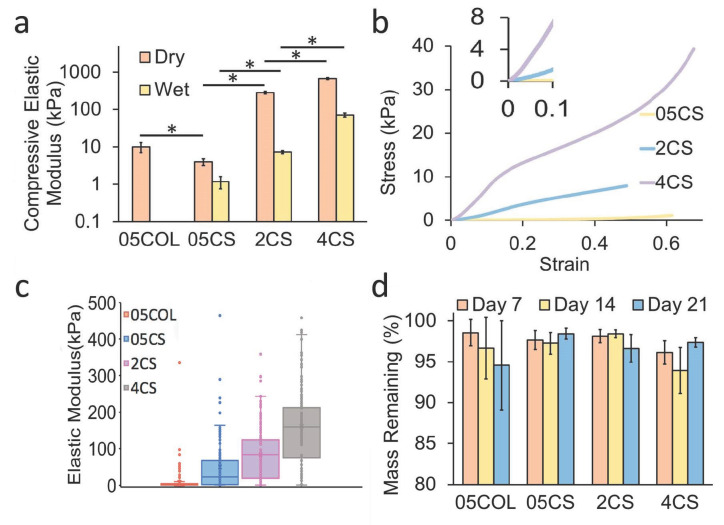
Mechanical properties of the scaffolds. (**a**) Compressive modulus of the scaffolds in dry and wet conditions (*n* = 5). (**b**) Stress–strain curves of the scaffolds in a wet condition. The 05COL scaffold was too soft to be measured in a wet condition. (**c**) Elastic modulus of the scaffolds in a wet condition. (**d**) Degradation of the scaffolds in a dynamic condition. Degradation rate analysis was performed by comparing the initial weight of the dry scaffolds with the dried weight of the scaffolds after incubation. There are no significant changes in weight after 21 days of incubation (*n* = 4). The differences between the samples are considered significant when marked with * based on Tukey’s test (*p* < 0.05).

To understand the microenvironment provided by the scaffolds, their potential influence on cell mechanosensing, and their ability to withstand agitation in dynamic cultures, a detailed analysis of their mechanical properties was conducted. This analysis encompassed both dry scaffolds and those soaked in PBS. The compressive elastic moduli (Figure 4a) of dry chitosan scaffolds exhibited an increasing trend from 4.1 ± 0.9 kPa to 284.5 ± 22.9 kPa and 681.9 ± 43.0 kPa, corresponding to the increasing chitosan concentration of 05CS, 2CS, and 4CS, respectively. Dry collagen scaffolds possessed a compressive elastic modulus of 10.0 ± 0.008 kPa. Considering that the cell culture was conducted in a medium while the scaffolds were hydrated, compression tests were also performed on “wet” scaffolds. In the hydrated state, the elastic moduli were 1.2 ± 0.4 kPa, 7.4 ± 0.7 kPa, and 71.2 ± 9.3 kPa for 05CS, 2CS, and 4CS scaffolds, respectively. However, wet 05COL scaffolds could not be evaluated through compression tests as they became excessively soft upon hydration. The elastic moduli of both dry and wet 2CS and 4CS scaffolds were higher than those of dry collagen scaffolds [36]. Representative stress–strain curves of compression tests are presented in Figure 4b, demonstrating an increase in ultimate compressive strength with a higher chitosan concentration. These results suggest that chitosan scaffolds may serve as superior protective structures for hNSCs compared to collagen scaffolds in dynamic cultures where microcarriers endure compression and shear forces.

To evaluate the micro- to nanoscale elastic moduli of wet scaffolds, AFM analysis was employed (Figure 4c). This analysis allowed for the characterization of mechanical properties relevant to mechanosensing. While compression tests address macroscale structural integrity and the resistance of pore walls to buckling, AFM tips measure intermolecular attraction and the resistance of chitosan or collagen polymer chains on the interior surface of the pore walls. The 05COL scaffolds exhibited the lowest average elastic modulus (3.7 ± 17.9 kPa), whereas the values for chitosan scaffolds increased from 50.1 ± 123.9 kPa to 83.2 ± 62.3 kPa and 160.9 ± 121.5 kPa as the chitosan concentration increased from 05CS to 2CS and 4CS, respectively. The localized compression, tension, and nano- or microscale features of the substrate play a crucial role in regulating cell fate through cell transmembrane receptors, ultimately influencing cytoskeleton and cell morphology. In our previous study, we investigated the impact of different degrees of deacetylation on the mechanical properties of chitosan films [67]. We found that a higher degree of deacetylation led to a decrease in the elastic modulus of the films. However, we also observed that this higher degree of deacetylation resulted in enhanced cell attachment, proliferation, and multipotency. Furthermore, increasing the molecular weight of chitosan led to an increase in the scaffold’s elastic modulus. This effect can be attributed to the entanglement of polymer chains [68].

In the subventricular zone and hippocampal dentate gyrus, where adult hNSC populations reside in humans and rodents, the reported elastic modulus is approximately 3 kPa [69,70]. However, there exists a considerable variation in measurement methods and results. Furthermore, the optimal elastic modulus for hNSC substrates remains uncertain, given the diverse outcomes observed in cell responses to softer substrates despite the success of conventional culture protocols on rigid plasticware. In this study, scaffolds with a wide range of mechanical properties were utilized to better assess the effect of these properties on cell fate. Mechanical properties, choice of materials, and resulting substrate surfaces collectively exert a coherent influence on cell responses.

### 3.4. Stability and Degradation in Culture Medium

Understanding the stability and degradation characteristics of scaffolds is crucial for designing effective tissue engineering strategies. To evaluate the stability of the scaffolds, their weights were monitored over a three-week period in a culture medium under rotation at 37 °C. Both chitosan and collagen have been shown to degrade in vitro and in vivo, with degradation rates depending on the specific conditions. Collagen, a widely used scaffold material, completely degrades after five weeks in a static culture medium [71]. Chitosan, on the other hand, can undergo partial depolymerization through hydrolysis within a few days when subjected to dynamic conditions in a medium [72]. It is worth mentioning that crosslinkers, such as genipin, can be added to the scaffolds to enhance mechanical properties and structural stability; however, they can also increase the autofluorescence of the scaffolds, making it difficult to image the cells [73].

In our study, all the scaffolds produced did not exhibit significant deterioration or degradation over the course of 21 days. Differences in the data may be due to scaffold-to-scaffold variability. They maintained over 90% of their weight after this period (Figure 4d). However, the 05COL scaffolds showed more variability and experienced a greater decrease in weight compared to those of the chitosan scaffolds. We expect our scaffolds to perform similarly through prolonged durations; the cells may also leave behind proteins that could adhere to the scaffold walls. These findings indicate that chitosan scaffolds are more durable in dynamic cultures, suggesting their suitability for long-term culture applications.

### 3.5. Maintenance of hNSCs in Dynamic Cultures

**Figure 5:**
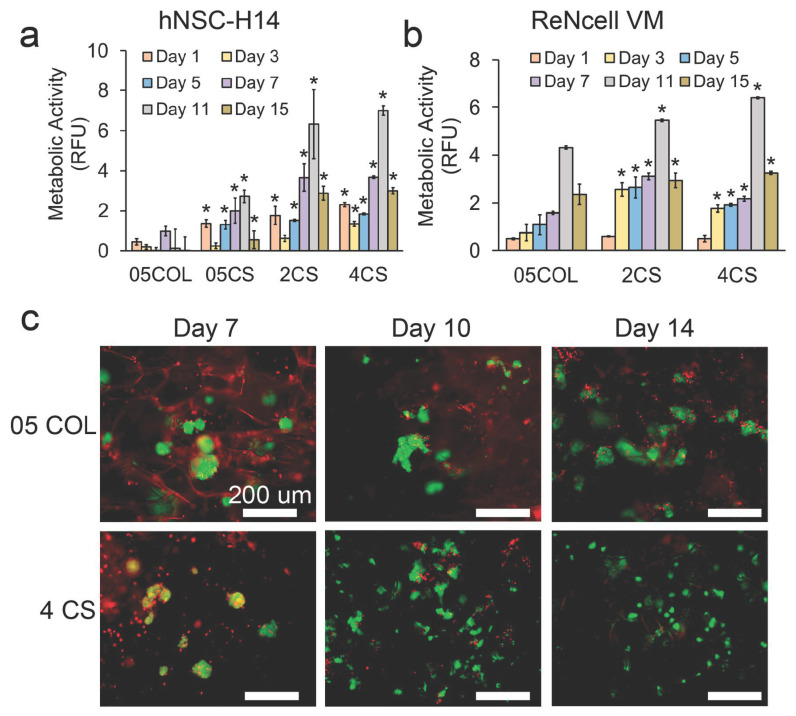
Assessment of cell proliferation rates and cell fate in hNSC-H14 and ReNcell VM cell lines using alamarBlue and live/dead stain. (**a**) Metabolic activity of hNSC-H14 measured by alamarBlue assay. (**b**) Metabolic activity of ReNcell VM measured by alamarBlue assay. (**c**) Fluorescent images of live (green) and dead (red) cells stained with calcein-AM and ethidium homodimer-1, respectively. Scale bars represent 200 µm. An * means that the difference between that sample and the respective 05COL sample is significant based on Tukey’s test.

We conducted dynamic culture experiments using porous scaffolds as microcarriers to support the growth of two different hNSC lines, namely hNSC-H14 and ReNcell VM. Initially, the cells were seeded onto the scaffolds in static well plates for one day and then transferred into dynamic culture vessels. The growth and proliferation of the cells were assessed using an alamarBlue viability/metabolic activity assay. The metabolic activity of hNSC-H14 and ReNcell in the scaffolds showed different trends (Figure 5a,b). For hNSC-H14, the metabolic activity in chitosan scaffolds showed a slight decrease on day 3 of the culture, followed by consistent increases until day 11. However, it decreased again on day 15, likely due to growth arrest caused by cell confluency. In contrast, cells in the 05COL scaffolds exhibited a modest increase on day 7 and overall limited growth after 15 days compared to those in chitosan scaffolds. As for ReNcell, the metabolic activity steadily increased in all scaffolds and reached its peak on day 11, followed by a decline on day 15, possibly due to confluency related factors. To accommodate longer culture periods, the initial cell seeding density could be decreased. Notably, the 4CS scaffolds demonstrated the highest overall metabolic activity for both hNSC-H14 and ReNcell. Consequently, the 05COL scaffolds exhibited restricted cell adhesion, which likely contributed to poorer activities compared to those in the 4CS scaffolds. Furthermore, the larger pore diameter of the 05COL and 05CS scaffolds might have failed to adequately protect or retain hNSC colonies.

To visualize cell adhesion and proliferation, the scaffolds were stained with calcein-AM and ethidium homodimer-1. Figure 5c illustrates the stained live and dead cells in the scaffolds, focusing on the hNSC-H14 cell line. As the culture progressed, the cells in the chitosan scaffolds exhibited more extensive expansion compared to that in collagen scaffolds. Moreover, the cells in the 4CS scaffolds displayed polarized morphology more frequently than those in the 05COL scaffolds did, suggesting a stronger adhesion to the 4CS scaffolds. Conversely, in the 05COL scaffolds, the cells formed larger clusters due to the preference for intercellular adhesion rather than cell–collagen interactions. Previous studies have reported that the increased surface positive charge resulting from the higher amino group content of chitosan promotes cell adhesion [74]. This favorable adhesion to chitosan scaffolds could further enhance cell proliferation [74].

**Figure 6:**
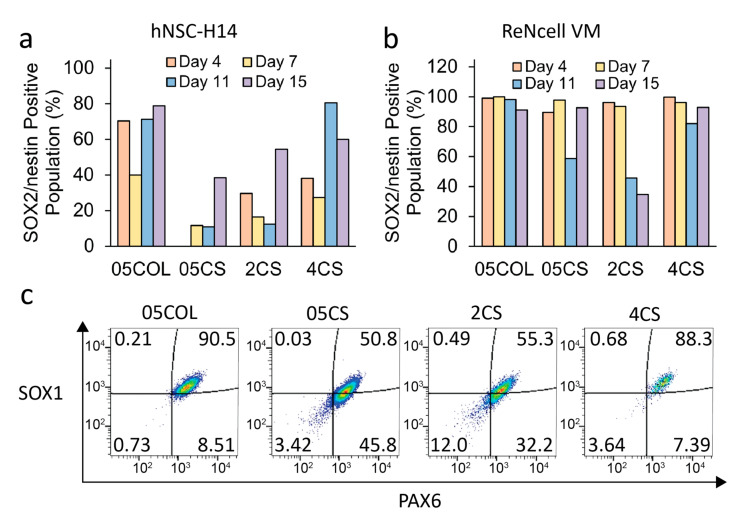
Flow cytometry analysis of cells collected from both cell lines over a 15-day period. Protein expression levels assessed for (**a**) hNSC-H14 and (**b**) ReNcell. (**c**) Dot plots representing the expression of SOX1 and PAX6 in ReNcells on day 7. Red color represent higher density, while blue color represent lower density.

Cells were harvested from the scaffolds over a period of 15 days, fixed, and then prepared for flow cytometry analysis. The multipotency of the cells collected from the microcarriers was assessed by staining for two crucial NSC protein markers, SOX2 and nestin. The time course of the populations positive for SOX2^+^/nestin^+^ in both cell lines is depicted in Figure 6a,b. Figure 6c illustrates the cell populations gated for SOX1^+^/PAX6^+^ expression in ReNcells after seven days of culture, representing additional NSC protein markers. The 05COL scaffolds exhibited the highest populations of both SOX2^+^/nestin^+^ and SOX1^+^/PAX6^+^ cells, while among the chitosan conditions, the 4CS scaffolds showed the most favorable results. Notably, hNSC-H14 demonstrated the highest percentage of SOX2^+^/nestin^+^ population on the 11th day of culture, which correlated with the metabolic activity data. Moreover, the porous scaffolds with large specific surface areas allowed hNSCs to proliferate for a longer duration compared to that with conventional 2D protocols while preserving their proliferative and multipotent state. Typically, in regular 2D culture protocols, hNSCs require passage every 3–5 days upon reaching 90–95% confluency to prevent growth arrest and spontaneous differentiation [75]. The growth kinetics and time course analysis of NSC proteins indicated that, in this study, the cells needed to be detached from the porous chitosan scaffolds before the 11th day of culture to ensure effective expansion of the entire population. However, despite having the narrowest specific surface area and porosity, the 4CS scaffolds achieved the highest yield of hNSCs, suggesting a potential protective effect against shear stress in a bioreactor. Stem cells possess mechanisms to sense mechanical stress, and the flow generated by agitation can induce cell death [75,76]. The higher compressive modulus of the 4CS scaffolds, compared to that of other chitosan scaffolds, may have shielded hNSCs from shear stress under dynamic conditions.

**Figure 7:**
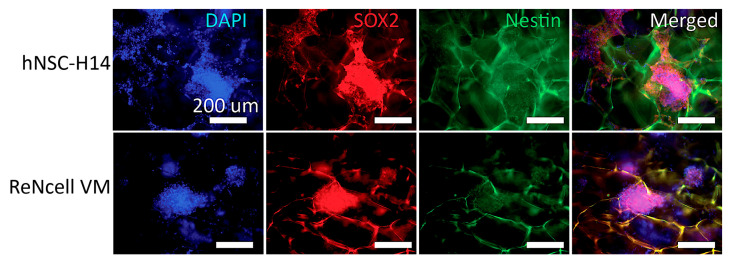
Immunofluorescence staining of hNSCs for SOX2 (red), nestin (green), and DAPI (blue) on the 4CS scaffolds after 3 weeks for hNSC-H14 (**top**) and ReNcell VM (**bottom**). Scale bars are 200 µm.

Further investigation into longer culture periods, lasting up to three weeks, was conducted. Immunostaining for SOX2 and nestin (Figure 7) revealed that the cells were able to sustain expression of these crucial NSC marker proteins for an extended duration compared to that with regular 2D controls. Over the three-week culture period, hNSCs cultured on 4CS scaffolds formed adherent aggregates within the scaffold pores. SOX2 localization was predominantly observed in the nucleus, although some translocation into the cytoplasm was evident, indicating partial differentiation resulting from overcrowding within the pores. At this stage, some aggregates reached confluency, occupying the pores without exceeding the desired size range of 300–500 µm in diameter to prevent necrosis.

Chitosan has been reported to promote the proliferation and differentiation of neurons and astrocytes [76]. However, its impact on hNSC maintenance has shown inconsistent results. In this study, multipotent hNSC colonies were successfully maintained in chitosan porous scaffolds optimized for use as microcarriers in dynamic cultures for up to three weeks. The chitosan scaffolds demonstrated their feasibility for long-term expansion of hNSCs in bioreactors under xeno-free and chemically defined culture conditions.

## 4. Conclusions

In this study, we developed a 3D microcarrier designed to expand hNSCs in dynamic bioreactor culture systems. The microcarrier offers several advantages, such as providing additional growth space and shielding cells from the dynamic environment. Our investigation into the maintenance of hNSCs in dynamic cultures showed that the mechanical properties of the scaffolds, including their compressive elastic moduli and localized micro- to nanoscale elastic moduli, have a crucial impact on cell mechanosensing, structural integrity, and cell fate determination. By utilizing pure chitosan, we successfully achieved xeno-free cultivation of human stem cells suitable for clinical applications. Notably, this is the first study to expand hNSCs using chitosan porous scaffolds in a dynamic culture system. The scaffolds maintained a stable microstructure and demonstrated long-term durability without degradation. The scaffold pores facilitated hNSC adhesion and distribution within a 3D space while possessing appropriate mechanical properties to support undifferentiated proliferation. When compared to collagen scaffolds, the optimized chitosan scaffolds outperformed them in maintaining the proliferation and multipotency of hNSCs. Moreover, the 4CS chitosan scaffolds were the best in supporting hNSCs in terms of proliferation and multipotency and were able to support hNSC expansion for up to three weeks. Overall, this study presents a dynamic bioreactor culture platform utilizing chitosan scaffolds as microcarriers capable of scaling up for hNSC production.

## Data Availability

The data presented within this study are available upon request form M.Z.
